# Trauma in the Kashmir Valley and the mediating effect of stressors of daily life on symptoms of posttraumatic stress disorder, depression and anxiety

**DOI:** 10.1186/s13031-019-0245-6

**Published:** 2019-12-12

**Authors:** Tambri Housen, Annick Lenglet, Showkat Shah, Helal Sha, Shabnum Ara, Giovanni Pintaldi, Alice Richardson

**Affiliations:** 1 National Centre for Epidemiology and Population Health, Australian National University, cnr Mills and Eggleston Rd, Canberra, ACT 2600 Australia; 2 Médecins Sans Frontières, Plantage Middenlaan 14, 1018 Amsterdam, DD Netherlands; 3 Radboud UMC, Nijmegen, The Netherlands; 4 Department of Psychology, University of Kashmir, Hazratbal, Srinagar, Jammu and Kashmir 190006 India; 5 Médecins Sans Frontières, Srinagar, Kashmir 190006 India

**Keywords:** Mediation, Stressors of daily life, Depression, Anxiety, Posttraumatic stress disorder, Conflict, Trauma

## Abstract

**Background:**

The negative psychological impact of living in a setting of protracted conflict has been well studied, however there is a recognized need to understand the role that non-conflict related factors have on mediating exposure to trauma and signs of psychological distress.

**Methods:**

We used data from the 2015 Kashmir Mental Health Survey and conducted mediation analysis to assess the extent to which daily stressors mediated the effect of traumatic experiences on poor mental health outcomes. Outcomes of interest were probable diagnosis of anxiety, depression, or PTSD; measured using the pre-validated Hopkins Symptoms Checklist (HSCL-25) and the Harvard Trauma Questionnaire (HTQ).

**Results:**

Total effect mediated were statistically significant but the proportions of effect mediated were found to be small in practical terms. Financial stress mediated 6.8% [95% Confidence Interval (CI) 6∙0–8∙4], 6.7% [CI 6.2–7∙7] and 3.6% [CI 3∙4–4∙0] of the effect of experiencing multiple traumaticogenic events on symptoms of anxiety, depression and PTSD, respectively. Family stress mediated 11.3% [CI 10.3–13.8], 10.3% [CI 9.5–11.9] and 6.1% [CI 5.7–6.7] of the effect of experiencing multiple traumatogenic events on symptoms of anxiety, depression and PTSD, respectively. Poor physical health mediated 10.0% [CI 9.1–12∙0], 7.2% [CI 6.6–8.2] and 4.0% [CI 3.8,4.4] of the effect of experiencing more than seven traumatic events on symptoms of anxiety, depression and PTSD, respectively.

**Conclusion:**

Our findings highlight that not only do we need to move beyond a trauma-focussed approach to addressing psychological distress in populations affected by protracted conflict but we must also move beyond focussing on daily stressors as explanatory mediators.

## Background

In recent years, there has been a move away from a trauma-focussed view of psychological well-being in populations affected by political conflict and towards a more holistic psychosocial understanding of risk factors for adverse mental health outcomes [1–5]. Conflict not only exposes a population to traumatic violent events but also impacts negatively on the social and material fabric of society. Survivors of violence associated with conflict are often concurrently subject to other stressors affecting everyday life and livelihood. The recognition of the mediating effect of daily stressors on the relationship between exposure to war trauma and mental health is increasingly reported in the literature [2, 5, 6], however, more evidence is needed exploring specific risk factors and from a variety of contexts.

Miller and Rasmussen (2010) [2] challenged the earlier assumption that the level of exposure to war related traumatogenic events determined severity of mental distress in conflict affected populations by demonstrating that daily stressors act as a partial mediator. Drawing on Kubiak (2005) [7] they argued that the chronicity of daily stressors has the potential to deplete coping mechanisms, therefore directly affecting the capacity to cope with traumatogenic events, and increasing the likelihood of these events leading to symptoms of mental distress.

Following the partition of India in 1947, the Kashmir Valley has been subject to continual political insecurity and ongoing conflict [8]. In 1989, an insurgency began leading to the displacement of over 100, 000 Kashmiri Pandits and 27 years of militant and military activity [9]. By 2015, approximately 70,000 Kashmiris had lost their lives in the conflict and 8000 people had been reported missing [10]. Frequent confrontations with violence have been reported including displacement, exposure to crossfire, ballistic trauma, round-up raids, torture, rape, forced labour, arrests/kidnappings and disappearances [11, 12]. The United Nations High Commission for Human Rights (UNCHR) 2018 report states that an estimated 130–145 civilians were killed by security forces between July 2016 and March 2018 and a further 16–20 killed by armed groups during the same period [13]. UNCHR estimate 1000 people were detained under the Armed Forces Special Powers Act (AFSPA, 1990) and the Jammu and Kashmir Public Safety Act (PSA, 1978) between March 2016 and August 2017, including minors [13]. These Acts, unique to the state of Jammu and Kashmir, create structures that promote impunity and injustice. The loss of human life, human rights abuses and a resulting context of ongoing low-grade conflict has had its impact on Kashmir’s population. In addition, the effect of prolonged exposure to violence on the psychological well-being of the population has been confounded by natural disasters such as a 7.6 Mw magnitude earthquake in 2005 and floods in 2014 [14–17]. In Kashmir other confounders include widespread poverty, uncertainty, grief, oppression and fear in addition to high unemployment with limited development of employment generating sectors [18].

The annual economic survey conducted by the Government of Jammu & Kashmir consistently report high rates of unemployment, 24.6% with a youth unemployment rate of 13.2% [19]. Employment-generating sectors such as commercial agriculture, forestry, fisheries and floriculture have been curtailed due to the prevailing political circumstances in the region. Where tourism was once the source of employment and economic growth, in the past 27 years it has become fractured and unreliable. In 2011, a Kashmir-based report by Mercy Corps [18] reported risks associated with high youth unemployment, which included feelings of failure, isolation, lack of social status, delayed marriages and an increase in tensions among disenfranchised young people, all of which have been compounded by the impact of future uncertainty related to ongoing political conflict. Expressions of disappointment, anger and hopelessness in addition to conflict-related stress, mental illness, suicide and drug addiction have been reported as prevalent in Kashmir’s youth [18].

Available mental health services in the Kashmir Valley follow a western biomedical model of care and treatment. Services are largely centralised in the main city of Srinagar. There is one dedicated psychiatric hospital, the Institute of Mental Health and Neurosciences (IMHANS), which provides inpatient and outpatient care. Other major hospitals in Srinagar also offer psychiatric services, with a few psychiatrists operating private clinics. Decentralised services are limited to a pool of Kashmiri psychiatrist and psychologists rostered to hold outpatient clinics at some of the district hospitals at set days of the week. The World Health Organization (WHO) has strongly advocated for the introduction of mental health in primary healthcare [20, 21] with research reporting successful implementation of primary care mental health programmes [22, 23]; however, few primary care workers know how to recognise an individual with mental health issues. In 1999, the government of India initiated the District Mental Health Plan (DMHP) with the intention of staggering a rolling out of community-based mental health services in all states of India [24]. The programme commenced in Jammu/Kashmir in 2004–2005, however, the 2012 National Mental Health Plan (NMHP), report results from a review of the DMHP stating it was barely functional in most districts [25]. The 2012 NMHP suggested a renewed commitment by the government of India to address the mental health needs of its population and calls for research which can ‘offer insights as well as pathways for change’. [26]

The majority of mental health studies conducted in the Kashmir Valley were conducted over ten years ago, however they consistently report a high prevalence of traumatogenic experiences and associated symptoms of mental distress. Khan (2013) measured mental health outcomes in 390 probability sampled urban households in four administrative regions of Srinagar, reporting that 46% of the sample suffered from anxiety and 32%, depression [27]. Between 2003 and 2005, Margoob et al. [28] used clinical interviews conducted by psychiatrists to assess the prevalence of PTSD in 2391 probability sampled individuals from six districts of the Kashmir Valley. Prevalence of PTSD was found to be 7% with a life-time prevalence rate of PTSD reported at 15% [28]. Using the Self Reporting Questionnaire (SRQ) and probability sample of 510 households in two districts in the Kashmir Valley in 2005, De Jong et al [11, 12] reported that psychological distress was experienced by 33% of their sample, with one-third reporting suicidal ideation. Research has also been conducted on the negative impact of natural disasters on mental health in the Kashmir Valley [14, 17, 29]. In 2015 MSF partnered with the Department of Psychology at Kashmir University and IMHANS to conduct a population based survey on mental distress in all ten districts of the Kashmir Valley [30, 31]. The survey found a strong correlation between exposure to multiple traumatogenic events and symptoms of Major Depressive Disorder (MDD), Generalized Anxiety Disorder (GAD) and Posttraumatic Stress Disorder (PTSD) [31] Among the traumatogenic events witnessed or experienced, 47% had witnessed the violent death of someone they knew. Alarmingly, 12% of survey respondents reported having had thoughts of ending their life in the four weeks prior to the survey, indicative of high levels of mental distress in the population [32].

These findings only partially explain the parameters affecting psychological health in the Kashmir Valley. What is less clear is how daily stressors mediate trauma history to predict mental health outcomes. This study aimed to quantify the mediating effect of daily stressors on the observed relationship between exposure to traumatogenic events and symptoms of mental distress in the Kashmir Valley, India. We used the information collected during the above mentioned survey to quantify the mediating effect of problems of daily life on the relationship between trauma exposure and mental health outcomes (MDD, GAD and PTSD symptoms).

## Methods

### Study design and participants

This study is a retrospective analysis of data from the 2015 Kashmir Mental Health Survey. The 2015 Kashmir Mental Health Survey was a cross-sectional population based study of 5428 randomly selected individual’s ≥ 18 years of age to estimate the prevalence and predictors of anxiety, depression and PTSD in the Kashmir Valley. The survey sample was drawn from a representative selection of 399 villages across all ten districts in the Kashmir Valley. A description of methodological procedures related to sampling and data collection for the survey is presented elsewhere [31].

Ethics Approval was obtained from Médecins Sans Frontières Ethics Review Board (ERB) (ID 1516), the Government Medical College Srinagar ERB (ID 19/ETH/GMC/ICMR), and the Australian National University Human Research Ethics Committee (ID 2015/516).

### Procedures

The survey questionnaire was developed using an iterative process involving multiple methods of free-listing [33], focus group discussions [34] and input from an expert panel. This has been described in detail elsewhere (Housen, et al. 2018 [30]). The final questionnaire comprised of multiple modules, these included: the Household Demographics Questionnaire (HDQ) and a Personal Interview Questionnaire (PIQ). The HDQ, administered to the self-identified head of the household, included information on demographic characteristics, family history of psychological illness and the household’s dependence on other persons for living. The PIQ was administered to a single, randomly selected individual within the household ≥18 years of age. Individuals were asked questions on the following topics: additional demographic information, ability to function in daily life, self-reported physical health, problems of daily life, substance use, coping strategies and exposure to traumatic events. In addition, the Hopkins Symptom Checklist (HSCL-25) for anxiety and depression, and the Harvard Trauma Questionnaire-16 (HTQ-16) for posttraumatic stress disorder (PTSD) were administered. Prior to the survey a separate study was conducted by the research team to culturally adapt, translate and validate the HSCL-25 and HTQ-16 following a rigorous process based on the sequences recommended by Brislin [35], Van Ommeren [36] and Flaherty, et al. [37] See Housen et al. 2017 [31] for a description of the methodology and results of the instrument validation process. The HSCL-25 and the HTQ-16 are not diagnostic tools; diagnosis can only be confirmed via clinical interview with a psychologist or psychiatrist. Therefore, the term ‘probable case’ is used throughout for persons scoring above the screening instrument cut-off point, with the recognition that use of screening tools also captures individuals with sub-syndromal illness [38].

Daily stressors commonly endorsed in free-listing interviews [33] and focus group discussions [34] included not enough money/financial problems, life is too expensive, poor physical health of self, unemployment, family problems, poor health of others, family pressure, job security, workload/overloading, anger/aggression, domestic violence, social isolation, nothing to do – sitting at home, quarrelling with others, and substance abuse. For the purpose of this analysis we combined ‘not enough money’ and ‘financial problems’ into one variable ‘financial stress’. We also combined ‘family problems’ and ‘family pressure’ into one variable ‘family stress’. The concept of ‘family problems’ was explored further in focus group discussions as part of a separate qualitative study (results reported elsewhere) [39] and were associated with: socio-cultural factors included those related to family conflict or family ‘tension’, stress associated with the inability to meet socio-cultural expectations such as the early marriage of children and dowry system, the breakdown of socio-cultural norms, interpersonal conflict, and for younger Kashmiri’s the pressures associated with familial and societal expectations related to academic performance.

The traumatic events checklist was adapted from the Life Events Checklist (LEC) [40] by an expert panel. The LEC traumatic events includes natural disasters, conflict related trauma, traumatic life experiences such as accidents and life threatening illness or injury, sexual trauma and death. Prior to the survey, a technical working group extended the section on conflict related trauma to include specific traumatogenic experiences relevant to the Kashmir Valley context, including crackdowns, frisking, interrogation with threats to life, torture, disappearance of friends or family, loss of property or belongings, forced separation from family members and direct combat exposure such as militant or military attacks. Respondents reported on one of four categories per event; 1. personally experienced this event, 2. witnessed this event happening to someone else, 3. know of someone this happened to, 4. don’t know anyone this has happened to. In concordance with standard practice [41] the traumatogenic events responses were collapsed into a binary ‘experienced and/or witnessed’ or ‘not experienced and/or witnessed’. The number of types of traumatogenic events experienced and/or witnessed was then calculated for each respondent.

The HSCL-25 [42] is composed of ten items designed to assess symptoms of anxiety and 15 items assessing symptoms of depression. Rating is via a four-point Likert scale with categories of response being: ‘never or no’, ‘sometimes’, ‘often’, or ‘always’. Three scores are calculated from the responses; the depression score (the average of the 15 depression items), the anxiety score (the average of the ten anxiety items) and the total score (the average of all 25 items). Across various population groups, the total score has been shown to be highly correlated with severe emotional distress of an unspecified diagnosis [42]. Separately the anxiety items and depression items are consistent with the Diagnostic and Statistical Manual IV (DSM-IV) diagnosis of generalized anxiety disorder and major depressive disorder (MDD), respectively. The HTQ-16 [42, 43], is the fourth section of a larger instrument which addresses 30 trauma symptoms derived from the DSM-IV criteria for PTSD. It is often used in isolation as a screening instrument for symptoms of PTSD. The checklist is comprised of 16 items rated on a four-point Likert scale, similar to the HSCL-25. The DSM-IV PTSD score is calculated from averaging the scores, with a higher score suggesting an increased probability of PTSD [43].

### Statistical analysis

**Fig. 1:**
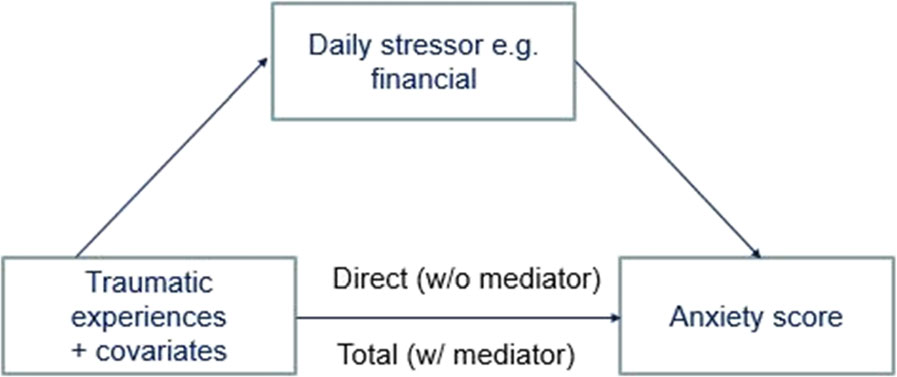
Conceptual model of the mediation relationship

We conducted mediation analysis to assess the extent to which any one of the fourteen daily stressors mediated the effect of traumatic experiences on outcomes (anxiety, depression, and PTSD). To enable comparison with previous work [31] we adjusted for gender, age (in 3 categories), education, marital status and employment status. Figure 1 provides an overview of the conceptualization of the mediation relationship.

A regression-based approach to testing for associations in mediation analysis is common [44]. Our approach used a continuous exposure, the number of types of traumatic events experienced. The outcome model used linear regression because the outcomes (scores on the HSCL25 and HTQ-16) are continuous. All analyses were carried out using Stata 15∙0 (Stata Corp 2017) using the medeff command of the mediation package [45]. All statistical tests were two-sided. Due to the large number of models and effects tested, 95% confidence intervals and *p* < 0.05 should be interpreted with caution with a view to the possibility of multiple comparisons leading to false positive results.

## Results

The demographic characteristics of survey respondents have been described elsewhere [31]. The majority of respondents were female, (64∙6%, *n* = 3509); overall mean age of respondents was 38.2 years (SD 15∙4 years) and 35∙1% (*n* = 1899) had received no education. Only 16∙3% (*n* = 886) were employed, and the majority of respondents (55∙3%, *n* = 3000) were undertaking ‘home duties’. Table 1 provides the median scores and mean scores of the outcome variables; the HSCL-25 has been separated into the subscales for depression (15 items) and anxiety (10 items), and for the exposure variable; the median and mean number of reported number of traumatic events is provided.

**Table 1:** Median and Mean scores on the HSCL-25 and HTQ-16 (*N* = 5428), Kashmir Mental Health Survey, 2015

Variable	Median (IQR)	Mean (SD, 95% CI)
Outcome variables		
HSCL- Anxiety subscale	1∙5 (1∙2–1∙9)	1∙50 (0∙48, 1.48–1.52)
HSCL- Depression subscale	1∙53 (1∙2–1∙93)	1∙58 (0∙51, 1.56–1.60)
HTQ-16-PTSD	1∙5 (1∙19–1∙88)	1∙55 (0∙41, 1.52–1.57)
Exposure variable		
Traumatogenic Events	7 (4–10)	7∙7 (4∙0, 7.52–7.93)

HSCL = Hopkins Symptoms Check List, HTQ = Harvard Trauma Questionnaire, PTSD = Posttraumatic Stress Disorder, SD = Standard Deviation, IQR = Interquartile range, CI = Confidence Interval

Table 2 presents the mediator variables alongside results of the mediation analysis, the percentage of the total effect of traumatogenic events on probable anxiety, probable depression and probable PTSD mediated by each mediator variable. Most mediators are statistically significant but the proportions of effect mediated are small in practical terms. The top three mediators of all three outcome measures of psychological distress were financial stress, poor physical health of self and family stress with proportions mediated ranging from 3.6 to 11.3%.

**Table 2:** Percent (0–100) of effect of multiple traumatogenic events on anxiety, depression and posttraumatic stress disorder mediated by daily stressors (95% CI), ordered by frequency of occurrence of daily stressor. Kashmir Mental Health Survey, 2015

	n (%)	Probable Anxiety % mediated (95%CI)	Probable Depression % mediated (95%CI)	Probable PTSD % mediated (95%CI)
*Financial stress*	3150 (58∙0)	6.8 (6.0, 8.4)	6.7 (6.2, 7.7)	3.6 (3.4, 4.0)
*Poor physical health of self*	2342 (43∙1)	10.0 (9.1, 12.0)	7.2 (6.6, 8.2)	4.0 (3.8, 4.4)
*Unemployment*	1437 (26∙5)	0.3 (0.3, 0.4)	0.8 (0.8, 1.0)	0.6 (0.6, 0.7)
*Family stress*	1419 (26.1)	11.3 (10.3, 13.8)	10.3 (9.5, 11.9)	6.1 (5.7, 6.7)
*Poor health of others*	1148 (21∙1)	2.4 (2.2, 3.0)	2.2 (2.1, 2.6)	1.4 (1.3, 1.6)
*Job security*	423 (7∙8)	−0.2 (−0.2, − 0.2)	1.0 (0.9, 1.1)	1.1 (1.0, 1.2)
*Workload*	368 (6∙8)	1.3 (1.2, 1.6)	1.0 (1.0, 1.2)	0.7 (0.7, 0.8)
*Anger/Aggression*	360 (6∙6)	1.6 (1.5, 2.0)	1.5 (1.4, 1.7)	1.2 (1.1, 1.3)
*Domestic Violence*	170 (3∙1)	2.7 (2.4, 3.2)	2.3 (2.1, 2.7)	1.1 (1.1, 1.2)
*Social isolation*	159 (2∙9)	0.3 (0.2, 0.3)	0.3 (0.3, 0.4)	0.2 (0.2, 0.2)
*Nothing to do- sitting at home*	149 (2∙7)	−0.03 (−0.04, −0.03)	−0.04 (− 0.04, − 0.03)	−0.02 (− 0.02, − 0.02)
*Quarrelling with others*	114 (2∙1)	1.9 (1.8, 2.3)	1.1 (1.0, 1.2	0.9 (0.9, 1.0)
*Substance abuse*	57 (1∙1)	0.02 (0.02, 0.03)	0.2 (0.2, 0.2)	0.3 (0.3, 0.3)

The percent mediated was higher for probable anxiety and smaller for probable depression and PTSD but the same top three mediators are found. The proportion of the total effect of experiencing multiple types of traumatogenic events on symptoms of anxiety, depression and PTSD mediated by financial stress was 6∙8%, 6∙7% and 3∙6%, respectively. Family stress mediated 11.3%, 10.3 and 6.1% of the effect of experiencing multiple types of traumatogenic events on symptoms of anxiety, depression and PTSD, respectively. The proportion of the total effect of experiencing multiple types of traumatogenic events on symptoms of anxiety, depression and PTSD mediated by poor physical health of self was 10∙0, 7.2 and 4.0%, respectively.

The inclusion of covariates did not alter the overall results in terms of the importance of financial stress, poor physical health of self and family stress (Additional file 1: Table S1). A moderation effect of each potential mediator was also tested (results not shown). Potential moderating variables that were consistent across models and outcomes were ‘unemployment’ and ‘job security’. However none of the effects were of sufficient practical significance to include here given the large number of models already reported.

## Discussion

This study explored whether daily stressors mediated the association between exposure to traumatogenic events and mental health in a setting of protracted political conflict. This is the first analysis of its kind on representative data on adverse mental health outcomes across all ten districts of the Kashmir Valley. Our results demonstrate only a minor mediating effect of a few commonly mentioned daily stressors; financial stress, poor physical health of self, and family stress. Thus providing only limited support to Miller and Rasmussen’s mediation model which suggests introducing daily stressors weakens the direct association between exposure to trauma and psychological distress [2].

In a prospective study conducted in Nepal comparing adverse mental health outcomes pre and post direct political violence, Kohrt et al. [46] demonstrated a dose-response effect of the number of conflict traumatogenic events on anxiety which persisted even after controlling for stressful life events and household income. In contrast, depression was not associated with war trauma. The authors suggest that risk factors for mood disorders may be more influenced by factors associated with poverty, age and gender. In previous analysis [31] older age, lower levels of education and being divorced, widowed or separated were significantly associated with adverse mental health outcomes in the Kashmir Valley; this could proffer some explanation as to the minor mediating effect of daily stressors and may point to dimensions of vulnerability as more significant risk factors for adverse mental health outcomes. The protracted conflict in Kashmir has led to an increase in the number of vulnerable groups including widows, orphans, and disabled [47]. In addition, high rates of unemployment and reported increased substance use by Kashmiri youth increase vulnerability [18, 19]. The association between psychological distress and vulnerability has been well established, and shown to be associated not only with socioeconomic factors [48] but also social exclusion mechanisms that are influenced by the conflict and redefined with the changing sociocultural landscape [49]. If vulnerability were to be a significant risk factor associated with adverse mental health outcomes then underlying levels of psychological distress could lead to overinflated estimates of psychological distress associated with trauma and potentially mask the extent of mediating effects of daily stressors.

Protracted conflict provides periods of peace interspersed with periods of political violence and insecurity. A recognized protective factor for improving mental health outcomes in populations affected by political conflict is the re-establishment of safety and security in the immediate environment [50, 51]. A large study conducted in Sri Lanka in 2009 found that persistent conditions of actual or perceived threat supported the genesis and maintenance of mental distress in the affected population [50]. Similarly, Silove et al. [51] reported an increase in post-traumatic stress disorder and severe distress related to recurrent violence in Timor-Leste. The continuation of violent flare-ups in the Kashmir Valley leave many in chronic heightened levels of stress, known locally as ‘*tension*’ or ‘*pareshani*’ due to the ‘*situation*’ or ‘*halat*’. Stress associated with the fear of the next act of violence has been discussed by Batniji et al. [52] and the continual exposure to stressful circumstances has been shown to increase psychological distress [53]. This may offer some explanation to the minimal mediating effect of daily stressors on mental health outcomes in our study as in fact the continual stress of potential acts of violence overtakes that associated with daily stressors.

A pre-occupation with injustice was also demonstrated by Silove et al. [51] to have a negative association with poor mental health outcomes. While we did not look at this relationship specifically in this study, a protracted history of human rights violations, lack of prosecution of perpetrators, a generalized community feeling of ‘occupation’ and impunity has been widely documented in the context of the Kashmir Valley both in the media and the literature [54–58]. The potential impact of preoccupation with injustice on mental health outcomes in the Kashmir Valley should not be underestimated and is worthy of further exploration.

A legacy of the impact of impunity is the intergenerational transmission of trauma and collective trauma, neither have been well studied in the context of protracted conflict. Largely limited to studies of Indigenous populations with traumatic histories [59] and families of Holocaust survivors [60], intergenerational trauma refers to the transmission of trauma across generations within families and communities, while collective trauma refers to psychological reactions to trauma that affect an entire society. Reported signs of intergenerational trauma include higher suicide rates and increased substance use, both of which have been reported in Kashmiri youth [18].

The main strengths of this study include the use of data derived from a large representative sample from all ten districts of the Kashmir Valley, the use of culturally adapted and validated tools to measure the outcome variables and the use of community-derived mediator variables. The use of the HSCL-25 allows for general cross-country comparisons, as it is a commonly used psychometric instrument in research in post-conflict settings.

Limitations of the study relate to the cross-sectional nature of the data and the interlinked nature of variables under analysis; socioeconomic variables such as education and employment, adverse mental health outcomes and exposure to traumatic life events prevents any assertion of causality. Although the dose-response association between the number of traumatogenic events experienced over a life-time and adverse mental health outcomes is well documented in the literature [61, 62], an additional limitation of this analysis was the equal weighting of traumatogenic events. Each type of traumatogenic event was counted as = 1 if it had been reported as experienced and/or witnessed; this approach did not recognize the relevance of heterogeneity in trauma exposure with the manifestation of symptoms of psychological distress. ‘Human rights violations’ have been shown to be predictors of PTSD, whereas ‘deprivation’ and ‘threat to life’ have been shown to be predictors of depression in older age [63]. For a more in-depth understanding of factors mediating the effect of trauma on adverse mental health outcomes in populations affected by political conflict a mixed-methods research design should be adopted where a qualitative and quantitative data are integrated to provide a more comprehensive understanding. A further limitation was that we tested mediators one at a time and did not pursue relationships between mediators. The small practical size of the mediating effects found, provides no evidence that the modelling would be enhanced by investigating multiple mediators in one model. The cross-sectional nature of the data also precludes ordering of mediators in time.

This study and many of those cited therein highlight the complexity of understanding the dynamic and inter-related nature of factors contributing to adverse mental health outcomes in populations affected by political conflict. Causality will continue to elude those who study mental health using cross-sectional studies and will challenge those who adopt more robust study designs. However, in spite of differences in the selection of psychometric instruments and other data collection tools, sampling methodology, contexts and research findings, researchers in this space all agree on the importance of the recognition of a holistic and comprehensive approach to mental health programming in populations affected by both ongoing and past political conflict.

## Conclusion

Mediation modelling has shown that amongst residents of the Kashmir Valley, financial stress, poor physical health of self and family stress were found to be the top three mediators of the relationship between multiple traumatogenic events and probable anxiety, depression and PTSD. However the percentages remain small, less than 14%. Our findings highlight that not only do we need to move beyond a trauma-focussed approach to addressing psychological distress in populations affected by protracted conflict but we must also move beyond focussing on daily stressors as explanatory mediators. Re-establishing safety and security, reducing vulnerability, strengthening the socio-economic environment and helping to rebuild socio-cultural practices must occur in combination if we are to mediate the impact of exposure of war-trauma on the psychology of affected populations. Other, less explored areas such as the impact of impunity, pre-occupation with injustice and intergenerational and collective trauma deserve greater focus in the context of protracted conflicts. This study adds to the empirical literature on the complexities of understanding psychological health in conflict-affected populations, in addition findings provide evidence for the targeting of mental health programs in the Kashmir Valley.

## Supplementary information

**Additional file 1: Table S1.** Percent (0–100) of effect of multiple traumas, adjusted for gender, age, marital status and employment status, on Anxiety, Depression and PTSD mediated by daily stressors (95% CI), ordered by frequency of occurrence of daily stressor. Kashmir Mental Health Survey, 2015.

## Data Availability

Access to data can be provided on reasonable request to and approval from the Medical Director at Médecins Sans Frontières, Amsterdam the Netherlands.

## Abbreviations

AFSPA: Armed Forces Special Powers Act
CI: Confidence Interval
DSM-IV: Diagnostic and Statistical Manual IV
ERB: Ethics Review Board
HDQ: Household Demographics Questionnaire
HSCL-25: Hopkins Symptoms Checklist, 25 items
HTQ: Harvard Trauma Questionnaire
IMHANS: Institute of Mental Health and Neurosciences
IQR: Interquartile range
LEC: Life Events Checklist
MSF: Médecins Sans Frontières
OHCHR: Office of the United Nations High Commission for Human Rights
PIQ: Personal Interview Questionnaire
PSA: Public Safety Act
PTSD: Posttraumatic Stress Disorder
SD: Standard Deviation
