# Supplementary material for: Trauma in the Kashmir Valley and the mediating effect of stressors of daily life on symptoms of posttraumatic stress disorder, depression and anxiety
Source: Confl Health. 2019 Dec 12;13:58. doi: 10.1186/s13031-019-0245-6 (PMC6909643; doi:10.1186/s13031-019-0245-6)
Supplement: Supplementary file 1 — Additional file 1: Table S1. Percent (0–100) of effect of multiple traumas, adjusted for gender, age, marital status and employment status, on Anxiety, Depression and PTSD mediated by daily stressors (95% CI), ordered by frequency of occurrence of daily stressor. Kashmir Mental Health Survey, 2015. [file 13031_2019_245_MOESM1_ESM.docx]

Supplementary Appendix: Re-establishing safety and security: priorities for addressing psychological distress in the Kashmir Valley

Supplementary Table 1 Percent (0 – 100) of effect of multiple traumas, adjusted for gender, age, marital status and employment status, on Anxiety, Depression and PTSD mediated by daily stressors (95% CI), ordered by frequency of occurrence of daily stressor. Kashmir Mental Health Survey, 2015.

|  | n (%) | Anxiety  % mediated (95%CI) | Depression  % mediated (95%CI) | PTSD  % mediated (95%CI) |
| --- | --- | --- | --- | --- |
| *Financial stress* | 3150 (58∙0) | 3.8 (3.4, 4.2) | 4.4 (4.0, 4.8) | 2.6 (2.4, 2.7) |
| *Poor physical health of self* | 2342 (43∙1) | 9.0 (8.1, 9.7) | 6.9 (6.3, 7.4) | 4.3 (4.0,4.6) |
| *Unemployment* | 1437 (26∙5) | 0.6 (0.5, 0.6) | 0.8 (0.7, 0.9) | 0.5 (0.5, 0.6) |
| *Family stress* | 1419 (26.1) | 10.2 (9.1, 11.0) | 10.6 (9.6, 11.4) | 6.2 (5.8, 6.6) |
| *Poor health of others* | 1148 (21∙1) | 1.5 (1.4, 1.7) | 1.4 (1.3, 1.5) | 1.1 (1.0, 1.1) |
| *Job security* | 423 (7∙8) | 0.2 (0.2, 0.3) | 0.7 (0.6, 0.7) | 0.7 (0.6, 0.7) |
| *Workload* | 368 (6∙8) | 1.0 (0.9, 1.1) | 1.0 (0.9, 1.1) | 0.6 (0.6, 0.7) |
| *Anger/Aggression* | 360 (6∙6) | 1.4 (1.2, 1.5) | 1.4 (1.3, 1.5) | 1.1 (1.1, 1.2) |
| *Domestic Violence* | 170 (3∙1) | 2.4 (2.2, 2.6) | 2.5 (2.3, 2.7) | 1.2 (1.1, 1.2) |
| *Social isolation* | 159 (2∙9) | 0.2 (0.2, 0.2) | 0.2 (0.2, 0.3) | 0.1 (0.1, 0.2) |
| *Boredom* | 149 (2∙7) | 0.1 (01, 0.1) | 0.1 (0.1, 0.1) | 0.1 (0.1, 0.1) |
| *Quarrelling with others* | 114 (2∙1) | 1.9 (1.7, 2.1) | 1.7 (1.5, 1.8) | 1.0 (0.9, 1.1) |
| *Substance abuse* | 57 (1∙1) | 0.1 (0.1, 0.1) | 0.1 (0.1, 0.2) | 0.2 (0.2, 0.2) |
